# The effects of the covid 19 pandemic on the elderly with depression

**DOI:** 10.1192/j.eurpsy.2021.1133

**Published:** 2021-08-13

**Authors:** I.D. Rădulescu, F. Sarbu, A. Ciubară

**Affiliations:** 1 Psychiatrist, “Elisabeta Doamna” Psychiatric Hospital, Galati, Romania; 2 Corresponding Author, Ph.d. Candidate, “Dunarea de Jos“ University, Galati, Romania; 3 Md, Ph.d., Hab. Professor, Faculty of Medicine and Pharmacy, University “Dunarea de Jos” Head of Psychiatry Department, Senior Psychiatrist at ”Elisabeta Doamna” Hospital, Galati, Romania

**Keywords:** Elderly, Depression, pandemic

## Abstract

**Introduction:**

Depression, as a psychiatric entity, has a number of emotional components. These are mainly known among patients over the age of 65: sadness, physical and mental exhaustion, irritability, feeling of emptiness and loneliness.

**Objectives:**

The main objective of this study is to detect if the effects of the Covid 19 pandemic over 65 years of age such as fear, excessive anxiety, lack of motivation, uncertainty and environmental changes, isolation (resulting in sleep disorders, appetite and attention) caused the exacerbation of depression.

**Methods:**

This study included a total number of 126 patients, each over 65, hospitalized at the Psychiatric Hospital “Elisabeta Doamna” in Galati in the context of the COVID-19 pandemic. They were diagnosed with depression, according to ICD-10 and the Hamilton scale.

**Results:**

All data obtained were centralized and used to detect whether, in Galați, the lockdown impacted the number of admissions of people over 65, diagnosed with depression, with an average age of 68,62. There is an increased incidence of female patients (75%), thus reporting an odds ratio of 3:1. The incidence of cases reported during the months of presentation is as follows: January (24%), February (28.8%), March (14.4%), April (3.2%), May (5.6%), June (23.2%).

**Conclusions:**

Environmental factors, unique in this situation, isolation, social distancing and changes in the daily routine, each associated with this global epidemiological crisis determinated a decrease of the number of depressive elderly admissions between March 15th and May 15th.

